# In situ decoration of Ag@exfoliated graphite composite catalyst for Fenton-like oxidation of methylene blue dye: kinetic and thermodynamic studies

**DOI:** 10.1186/s13065-025-01584-1

**Published:** 2025-07-24

**Authors:** Somia M. Abbas, Khadiga M. Abas

**Affiliations:** 1 Inorganic Chemistry Department, National Research Centre, 33 El Bohouth St., (Former El Tahrir St.), Dokki, Giza 12622 Egypt; 2 Physical Chemistry Department, Advanced Materials Technology and Mineral Resources Research Institute, National Research Centre, 33 El-Bohouth St, Giza, 12622 Egypt

**Keywords:** Exfoliated graphite (EG), Silver nanoparticles (AgNPs), Composite catalyst, Homogeneous Fenton-like catalytic degradation

## Abstract

Amendable nanomaterials are preferred for wastewater remediation. Silver (Ag) and graphene materials have drawn significant consideration for wastewater treatment due to their good dispersibility in aqueous systems, biocompatibility, and catalytic activity. For this purpose, an easy-to-follow procedure has been developed to prepare exfoliated graphite (EG) involving a binary-component system composed of concentrated H_2_SO_4_ and (NH_4_)_2_S_2_O_8_. Afterwards, Ag@EG composite catalysts were prepared by in-situ decoration of silver nanoparticles (AgNPs) over EG by sonication. The prepared catalysts were investigated for methylene blue (MB) dye removal from wastewater using a homogeneous Fenton-like oxidation process (H_2_O_2_/Ag^+^). Process-controlling factors like H_2_O_2_ dose, solution pH, and dye concentrations were explored. The results showed that MB dye degradation in the Fenton-like oxidation process can be clarified by a pseudo-first-order kinetic model. The highest dye degradation efficiency (99.8%) was achieved using Ag@EG (1:1). The morphology and structural characteristics of the prepared catalysts were evaluated by SEM, TEM, EDX, XRD, Raman spectroscopy, FTIR, and UV-Vis analyses. The activation energy, enthalpy, and entropy of the catalytic degradation of the MB dye in the presence of prepared catalysts were established. The data elucidated that most MB dye degraded within 120 min when using mesoporous Ag@EG (1:1) catalyst. Ag@EG (1:1), with a high S_BET_ (87 m^2^/g), functioned as a Fenton-like oxidation catalyst, exhibiting high degradation efficiency compared to other cited catalysts.

**Supplementary Information:**

The online version contains supplementary material available at 10.1186/s13065-025-01584-1.

## Introduction

Lately, the rapid expansion of the industrial sector has led to the release of hazardous effluents containing harmful materials [1]. Environmental and health organizations have raised concerns regarding wastewater, which contains dyes and pigments that affect aesthetic coloration despite an insignificant amount of discharge into the surroundings. The extreme color, high chemical oxygen demand (COD), and total organic carbon (TOC) of dyes threaten marine organisms and adversely impact the aesthetics of the environment [2]. The highest toxicity estimates were detected among basic and diazo direct dyes. Methylene blue (MB) is a water-soluble basic (cationic) dye. It is considered valuable in the leather, calico, cotton, and tannin industries, as well as for biological staining purposes [3]. Prolonged contact with MB dye may lead to gastrointestinal and ocular issues, as evidenced by symptoms such as nausea, vomiting, and diarrhea [4, 5]. Numerous techniques have been adopted for treating wastewater containing organic dyes, including biological treatment, adsorption [6], coagulation, and chemical oxidation [6]. Adsorption approaches are extensively used in this process because they are less costly, more successful at eliminating dyes from aqueous media, require fewer operational steps, and allow for the recycling of solid adsorbents [7]. Despite this, some contaminants are resilient to the standard physical and biological processes implemented in wastewater treatment plants because of their chemical features [8]. Therefore, advanced oxidation processes (AOPs) have been reported as practical, inventive, and effective alternatives to eliminate organic pollutants from water bodies [9]. These processes release species, essentially hydroxyl radicals (HO^•^), which attack and oxidize their target contaminants [9, 10]. The significant characteristics that set these processes ahead of others are their capacity to work in close proximity to ambient conditions, the nonselective nature of HO^•^ radicals, and the transformation of contaminated dyes into harmless products like CO_2_ and H_2_O [11]. The decomposition of many kinds of organic compounds in industrial wastewater has been demonstrated to be successful using one of the AOPs [12], homogeneous and heterogeneous phase chemical oxidation processes (H_2_O_2_/Fe^2+^ (Fenton’s reagent)) [13, 14]. When used as an oxidizing agent, H_2_O_2_ has potent qualities and can transform hazardous organic materials into less dangerous ones [15].

Fenton-based reactions are powerful AOPs. They utilize a catalyst, typically iron, along with hydrogen peroxide to create highly reactive hydroxyl radicals (•OH). These reactions are widely applied, particularly in environmental cleanup, due to their strong oxidizing capabilities. The standard Fenton reaction combines ferrous iron (Fe²⁺) with hydrogen peroxide (H₂O₂) [13, 14], primarily generating highly reactive hydroxyl radicals (•OH) by oxidizing ferrous iron to ferric iron (Fe³⁺), as shown in the reaction: Fe^2+^ + H_2_O_2_ → Fe^3+^ + •OH + OH^−^. Afterward, the ferric iron can be converted back to ferrous iron, which allows the catalytic cycle to continue (Fe^3+^ + H_2_O_2_ → Fe^2+^ + HO_2_•+ H^+^). This step produces a hydroperoxyl radical (HO_₂_•). Both hydroxyl and hydroperoxyl radicals are highly reactive and can participate in further reactions with organic pollutants or other species in the system [15].

Certain metallic nanoparticles or ions can enhance H_2_O_2_’s conversion into hydroxyl radicals, consequently improving its efficacy in pollutant degradation [16]. A unique combination of nanosensing [17–19], antibacterial activity [20, 21], and localized surface Plasmon resonance (LSPR) [18] makes silver nanoparticles (AgNPs) the most desirable metallic nanoparticles. Additionally, silver is more stable in an aqueous environment compared to less costly noble metals like copper or iron [22]. Numerous processes, including chemical reduction [23], photoreduction [24], pulse laser ablation [25], or biosynthesis [26, 27], have been reported to yield AgNPs with varying sizes and morphologies. Despite this, the chemical reduction approach, which employs a capping and reducing agent, is one of the most flexible synthesis methods [28]. Using this approach, stable AgNPs can be generated in water under ambient conditions with a minimal addition of a reducing agent (often NaBH_4_, which is already in use in the industry) [29]. The method uses water as a solvent, requires minimal reagents, and produces adaptable nanoparticles, making it environmentally beneficial even without heating.

Although AgNPs exhibit toxicity to a broad spectrum of pathogens, achieving antibacterial purposes, they show low cytotoxicity to human cells, making them applicable to biomedical and clinical fields [30, 31]. Numerous studies [32–34] have indicated that the toxicity observed with silver nanoparticles (AgNPs) is primarily attributable to the dissolution and subsequent release of silver ions (Ag^+^). However, the direct contribution of the nanoparticle itself to overall toxicity remains an area requiring further elucidation. Characterization and assessment of the fate and behavior of AgNPs depend on the test medium and species [35]. Recovering silver nanoparticle (AgNP) powders from water is challenging and can cause further contamination. So, extensive research and developmental initiatives have been undertaken to mitigate the deleterious effects of AgNPs in dynamic aqueous environments [36, 37]. M. Guo et al. succeeded in embedding AgNPs within porous oil-absorbent materials, which has proven to be an effective method for enhancing their recyclability [38].

AgNPs and other metal nanoparticles can be formed on a solid substrate in addition to liquid-phase synthesis. Metal nanoparticle dispersion can be supported by various carbon-based fillers, such as graphene oxide (GO), reduced graphene oxide (rGO), exfoliated graphene layers (EG), graphene flake (GF), and carbon nanotubes (CNTs) [39, 40]. However, GO and CNTs are not appropriate for large-scale and cost-effective applications due to their complicated preparation techniques and high assembly costs. Conversely, EG can be developed easily and affordably for widespread manufacturing, making it a good filler for polymer composite construction [41]. EG is typically produced by heating graphite intercalation compounds (GICs), generated through physical or chemical procedures, under a microwave or high temperature [42, 43]. The most popular techniques comprise using oxidants to break the edges of graphite layers. This mechanism enables intercalating agents to penetrate graphite interlayers by increasing the space between graphite layers [44]. We present an innovative and practical approach for producing EG in large quantities, where EG can be created in a single preparation stage at room temperature. The binary-component system, combining concentrated H_2_SO_4_ with ammonium persulfate ((NH_4_)_2_S_2_O_8_), has been a commonly utilized traditional production technique for EG. Surprisingly, it was discovered that native graphite introduced to the binary-component system at room temperature could transform into worm-like EG with minimal stirring and standing when the amount of concentrated H_2_SO_4_ was drastically reduced [45].

Our work demonstrates that EG can be obtained by a binary-component system comprising (NH_4_)_2_S_2_O_8_ and concentrated H_2_SO_4_ for native graphite flakes (GF). This is followed by the in situ incorporation of AgNPs onto the EG surface as a substrate, using a mixture of Na_3_C_6_H_5_O_7_ and NaBH_4_ as reducing agents to form Ag@EG (0.5:1, and 1:1) composite catalysts. To identify the morphology, crystal phases, and functional surface groups of the samples, we employed scanning electron microscopy, X-ray diffraction, Raman spectrum, Fourier transform infrared, and UV-Vis spectroscopic analyses. The present study provides insight into the violent reaction between zero-valent silver and hydrogen peroxide under controlled Fenton conditions, confirming the production of hydroxyl radicals by the reaction of prepared composite catalysts with H_2_O_2_. It also presents thermodynamic and kinetic models describing degradation ratios at varying temperatures and time intervals. Comparative studies on the Fenton-like catalytic degradation of MB dye demonstrate good performance when Ag@EG (1:1) was employed.

## Materials and methods

Graphite flakes (GF) (99% carbon basis, -325 mesh particle size) and AgNO_3_ were procured from Sigma-Aldrich. Concentrated H_2_SO_4_ (98%), HCl (35%), absolute ethanol, NaOH, and ammonium persulfate ((NH_4_)_2_S_2_O_8_, APS) were supplied by Merck Chemicals Co. Ltd. Hydrogen peroxide (H_2_O_2_, 30%) and sodium borohydride (NaBH_4_) were purchased from Fisher Scientific Co. Tri-sodium citrate (Na_3_C_6_H_5_O_7_) was obtained from LOBA CHEMIE PVT.LTD. The chemical structure of Methylene blue (MB) dye, acquired from Fisher Scientific Co., is shown in Fig. S1.

### Exfoliation of graphite flakes

An easy-to-follow procedure has been developed to prepare exfoliated graphite (EG) involving a binary-component system composed of concentrated H_2_SO_4_ and (NH_4_)_2_S_2_O_8_ (APS). First, APS and H_2_SO_4_ were combined in a 1:1 ratio and sonicated for 15 min at room temperature. After introducing 1 g of GF to the previously established mixture, a slurry consisting of the binary component solution and GF was obtained. Sonication was subsequently continued for an additional 15 min. After two hours of constant stirring in an 80 °C hot water bath to produce a more viscous solution, the mixture was allowed to cool for 30 min at room temperature before being centrifuged. After obtaining acidic EG, it was cleaned with distilled water until a neutral pH was attained, then dried for one hour at 60 °C in an air oven.

### In situ fabrication of Ag-based composite catalysts

The composites were synthesized via an in situ chemical reduction approach using EG as a support for Ag-based catalysts with two weight ratios (0.5:1 and 1:1) based on EG. The amounts of AgNO_3_ and EG corresponding to these weight ratios were calculated and weighed. AgNO_3_ and EG were then dissolved and dispersed, respectively, in two separate beakers containing 30 ml of absolute ethanol. The two solutions were mixed drop-wise and stirred for 30 min. The reduction of Ag^+^ ions to metallic Ag^0^ was achieved using a mixture of Na_3_C_6_H_5_O_7_ and NaBH_4_ as reducing agents. The synergistic effect of the reducing agent mixture prevents the accumulation of the produced AgNPs by acting as both reducing and stabilizing agents. The amounts of reducing agents were calculated using molar ratios of AgNO_3_:Na_3_C_6_H_5_O_7_ (2:1) and AgNO_3_:NaBH_4_ (1:1), according to Eqs. (1) and (2).

1 $$\eqalign{ 2{\rm{AgN}}{{\rm{O}}_3} + {\rm{N}}{{\rm{a}}_3}{{\rm{C}}_6}{{\rm{H}}_5}{{\rm{O}}_7} \to & 2{\rm{A}}{{\rm{g}}^0} + {\rm{N}}{{\rm{a}}_2}{{\rm{C}}_5}{{\rm{H}}_4}{{\rm{O}}_5} \cr & + {\rm{C}}{{\rm{O}}_2} + {\rm{NaN}}{{\rm{O}}_3} + {\rm{\;HN}}{{\rm{O}}_3} \cr}$$

2 $$\:\:{\text{A}\text{g}\text{N}\text{O}}_{3}+{\text{N}\text{a}\text{B}\text{H}}_{4}\to\:\:{\text{A}\text{g}}^{0}\:+\frac{1}{2}{\text{H}}_{2}\:+\frac{1}{2}\:{\text{B}}_{2}{\text{H}}_{6}\:+\:{\text{N}\text{a}\text{N}\text{O}}_{3}$$

The calculated amounts of the reducing agents were weighed and dissolved in distilled water. The mixture of reducing agents was then added drop-wise to the mixture of AgNO_3_ and EG with stirring. The mixture was protected from light by covering the reaction vessel with aluminum foil to prevent photochemical reactions. Finally, it was continuously dispersed using ultrasonic water bath, BRANSON 3510 (USA), equipped with a titanium tip at a frequency of 40 kHz and at 25ºC for 1 h. A “mirror-like silver layer” appeared on the vessel wall and solution surface, indicating AgNPs formation (Fig. S2). The final product was filtered, washed with absolute ethanol and distilled water, then dried and homogenized using freeze-drying equipment at − 20 °C. The resulting composite catalysts were labeled Ag@EG (0.5:1) and Ag@EG (1:1).

### Physicochemical characterization

Scanning electron microscopy (SEM) and energy dispersive X-ray analysis (EDX) (QUANTA FEG 250E-SEM, Japan) were applied to evaluate the morphology, and elemental content of the developed catalysts. The transmission electron microscopy (TEM) image was acquired using a JEOL JEM-2100 F microscope (Tokyo, Japan). This instrument was run at 200 kV and included a Cs corrector, allowing for atomic resolution better than 0.14 nm. The image was taken at a resolution of 200 nm. X-ray powder diffraction patterns (XRD) of the final samples were recorded using a Philips X’Pert apparatus equipped with a CuKα X-ray source (λ = 1.54056 Å). Data were collected in the 2*θ* range of 10–80° at a step of 0.05°. Raman spectra were recorded at room temperature using a WiTec, 300R alpha micro-Raman spectrometer (Germany) equipped with a confocal Raman microscope in the ViaTM system, with a 532 nm laser excitation. Fourier transform infrared spectroscopy (FTIR) measurements of the prepared samples were captured using the KBr pellet technique on a NICOLET 8700 spectrometer (Thermo Scientific, United Kingdom). The spectra were recorded across a range of 4000–400 cm^− 1^. UV-Visible absorption spectra of prepared samples were analyzed at room temperature using a Shimadzu UV-Vis spectrophotometer in the wavelength range of 200–500 nm. Dimethylformamide served as the solvent and reference. Pore characteristics and specific surface area of synthesized samples were determined through N_2_ adsorption-desorption experiments at -196 °C. These measurements utilized a Belsorp max version 2.3.2 analyzer (Microstac Retsch GmbH, Haan, Germany) and the Brunauer–Emmett–Teller (BET) approach.

### MB catalytic degradation

The MB dye decolorization process was performed in batches. Distilled water was used to prepare the dye solution. The Fenton degradation process was tested in a 500 mL glass beaker under different experimental settings in dark conditions: pH 2–6, dye concentrations (10–100 mg.L^–1^), oxidant dosage of 10–80 mM H_2_O_2_, and temperature of 298–318 K. A thermostat regulated the temperature. In each experiment, 250 mL of MB aqueous solution was incorporated with 0.25 g of each prepared catalyst. The pH was adjusted with either 0.1 M HCl or 0.1 M NaOH while the mixture was stirred. Oxidant doses were included to initiate reactions, and reaction time was observed. The dye solution’s absorbance was assessed and reported before and after each oxidative degradation. The remaining MB concentration was detected using the UV-Vis spectroscopic approach at 665 nm (UV-2401PC spectrophotometer) and a 1 cm quartz cuvette. Furthermore, to optimize reaction conditions, degradation efficiency was estimated using the subsequent equation:

3 $$\:\text{D}\:\left({\%}\right)=\left(1-\frac{\text{A}{}_{\text{t}}}{{\text{A}}_{^\circ\:}}\right).\:100$$

A_°_ and A_t_ symbolize the absorbance of the dye before and after (at time, t) Fenton reaction. To establish the kinetic and thermodynamic aspects of Fenton-like oxidation reactions, reactant mixtures containing MB (20 mg.L^− 1^) and optimal reaction settings at three different temperatures (298, 308, and 318 K) were repeatedly scanned at constant 10-minutes intervals. These scans demonstrate that the absorbance intensities at λ_max_ gradually decreased with time and at high temperatures, affirming the onset of dye degradation by the Fenton reagent.

## Results and discussion

### Microscopic analyses

**Fig. 1:**
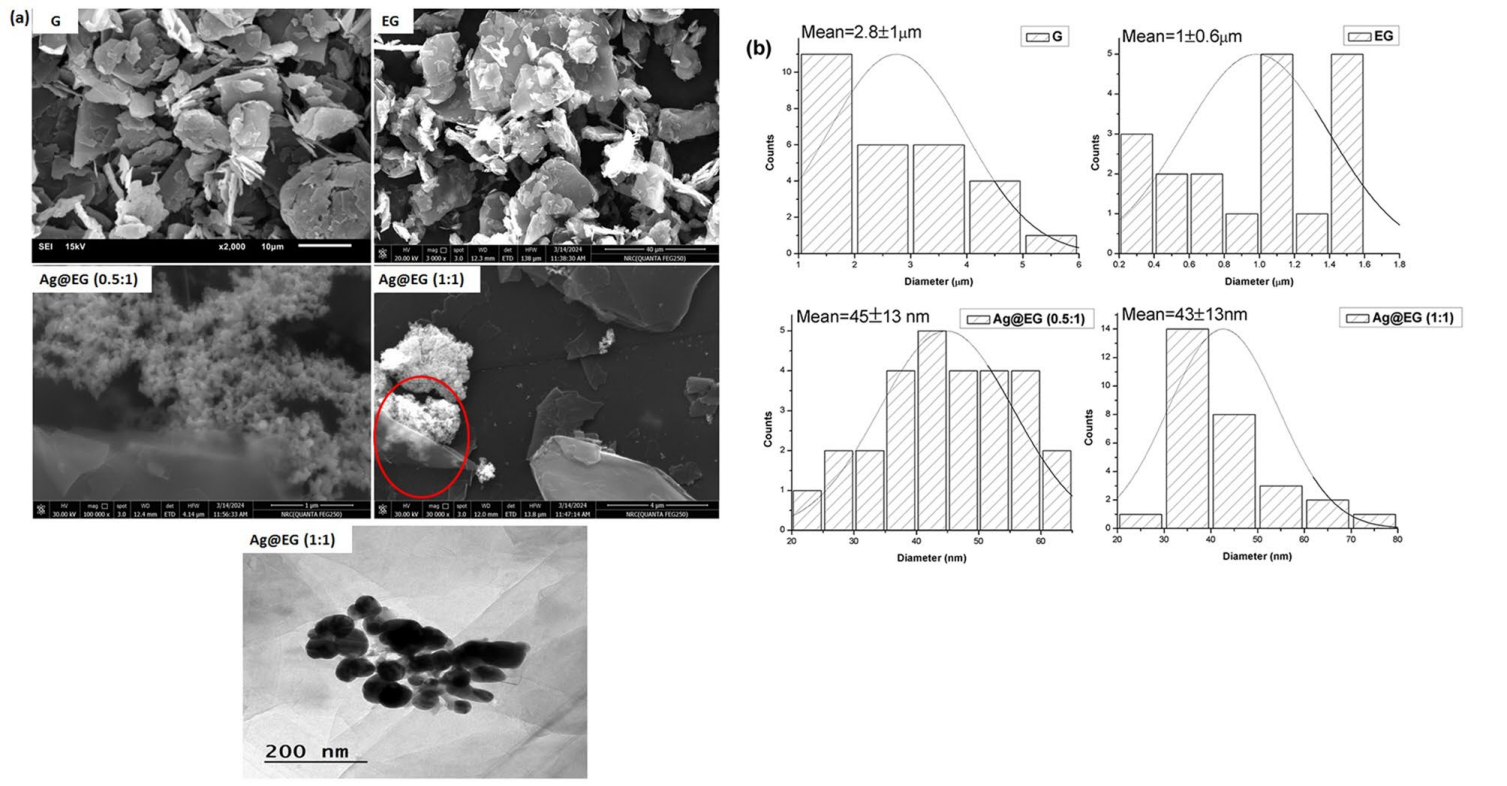
(**a**) SEM micrographs of prepared catalysts with TEM image of Ag@EG (1:1), and (**b**) Histogram of diameter distribution curves of prepared catalysts

**Fig. 2:**
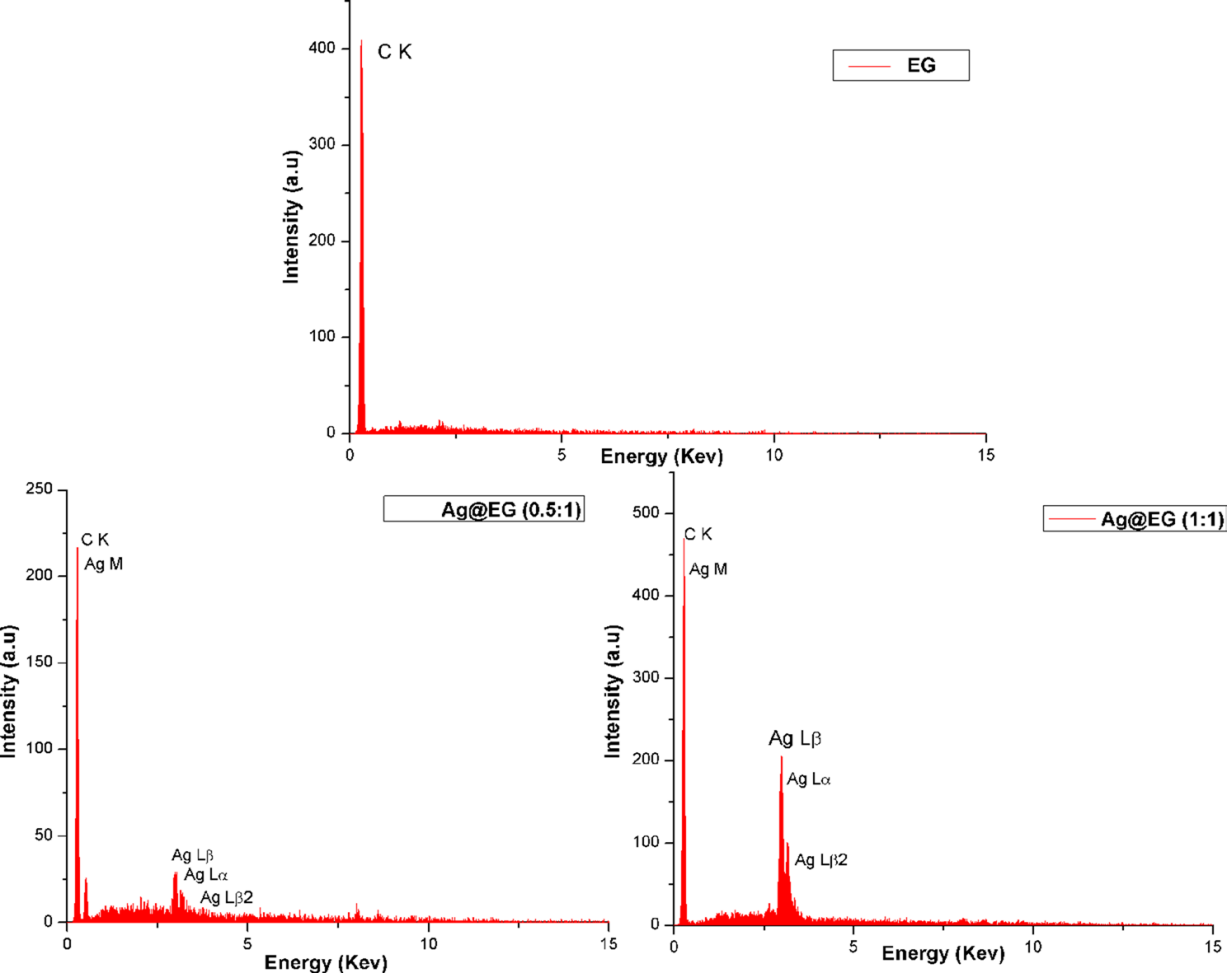
EDX spectrum of prepared catalysts

SEM, TEM, and EDX inspections were conducted to look into the morphological features of the developed samples; the results are exhibited in Figs. 1(a, b) and 2, respectively. The captured images of GF and its modified surface (EG) depict that the GF powder is composed of flake-like particles arranged in a densely stacked, regular sheet structure with a mean diameter of 2.8 ± 1 μm. Following hydrothermal treatment, the constituent graphene sheets undergo exfoliation, leading to turbostratic restacking and fracturing of graphite planes. This results in a disordered structure and some cracked surfaces with a drastically reduced diameter [46]. The interlayer of graphite sheets may be effectively penetrated by the exfoliating agent (NH_4_)_2_S_2_O_8_. Its dissolution can generate gases that exfoliate thick graphite flakes and surpass the Van der Waals forces between graphite layers. This structural change suggests a potential exfoliating impact of hydrothermal treatment [47]. The particle size distribution profile of EG also authorizes this size-decreasing phenomenon, exhibiting an average diameter of 1 ± 0.6 μm of EG particles. Figure 1(a, b) presents SEM images of as-prepared Ag@EG-based catalysts with two ratios (0.5:1 and 1:1) and particle size distributions of AgNPs, respectively. SEM micrographs (inside the red circle) verify the exfoliation of graphite into monolayer sheets, showing EG as a bright and transparent flake in the as-prepared composite catalyst. Additionally, the micrographs imply that EG was decorated with AgNPs, creating a cauliflower-like morphology of connected Ag metal framework clusters [39]. The assembly of some aggregates with distinct nucleation sites and the hierarchical growth of spherical AgNPs with diameter distributions of 45 ± 13 nm and 43 ± 13 nm for Ag@EG (0.5:1) and Ag@EG (1:1), respectively.

The TEM image of the Ag@EG (1:1) composite catalyst illustrates the spherical and circular nature of the AgNPs (dark spots), which are homogeneously attached on the EG sheets. The particles are mostly mono-dispersed and separated from each other, with no serious aggregation or fusion. This demonstrates the high stability of the Ag@EG composite and indicates the synergistic effect of both reducing and capping agents (NaBH_4_ and Na_3_C_6_H_5_O_7_) on the nucleation, growth, and stabilization of AgNPs, allowing the formation of small and spherical particles. The average size of the AgNPs was found to be 38 nm (which is comparable to that obtained from SEM). Figure 2 illustrates the elemental content observation of prepared catalysts. Treated EG exhibited 100% carbon content. However, after decoration with AgNPs, the carbon content of Ag@EG (0.5:1) decreased to 85%, with approximately 15% Ag content. Additionally, with an elevated silver ratio, the Ag content of Ag@EG (1:1) increased to 35%.

### Structural spectroscopic analyses

#### XRD analysis

**Fig. 3:**
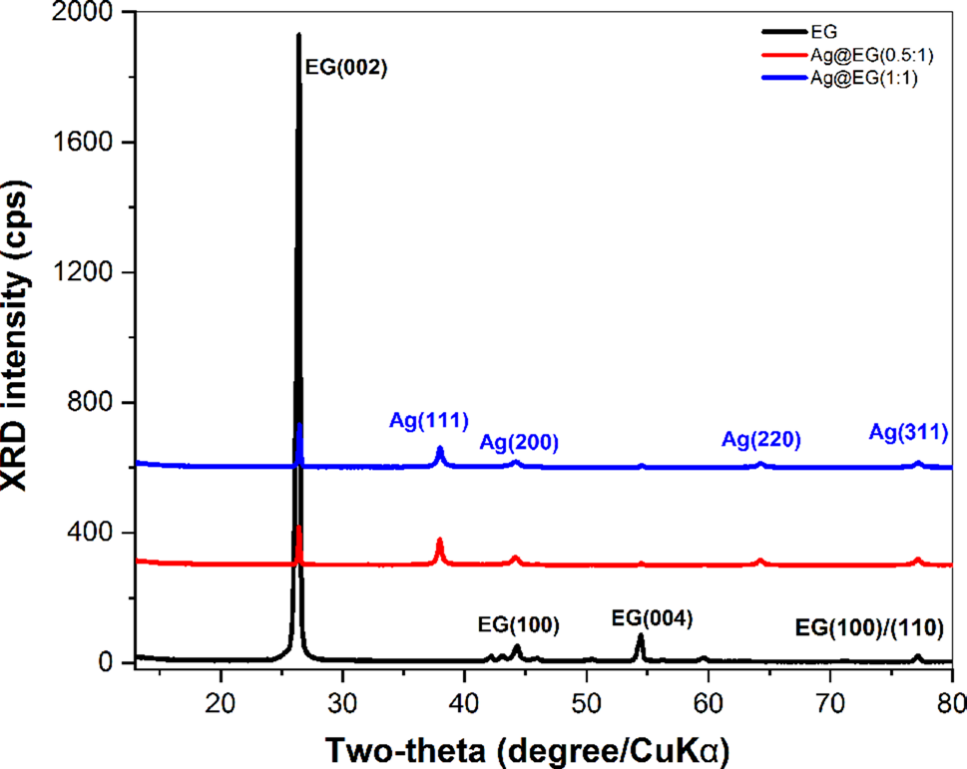
XRD patterns of prepared catalysts

X-ray diffraction (CuKα radiation) demonstrated the crystalline nature of the developed EG and its strong affiliation with AgNPs (Fig. 3). The diffraction patterns of EG and its composite catalysts illustrate that the EG powders contain a pure hexagonal graphite-2H phase, according to standard card PDF number 41-1487 [48]. The interplanar distance between graphitic basal planes is determined using the Bragg diffraction formula: nλ = 2d.sinθ, where λ is the X-ray wavelength (0.154 nm), d is the interplanar spacing, and θ is the Bragg angle. Strong EG characteristic patterns can be detected at 2θ = 26.4º (d-spacing of 3.37Å) and 2θ = 54.4º (d-spacing of 1.68Å) along the c-axis. These patterns correspond to the (002) and (004) planes, respectively. The sidewall of EG sheets is defined by the (002) diffraction peak. The small peak at 54.4° is attributed to the partial oxidation of carbon double bonds in graphite [39]. Sulfuric acid and APS, used during preparation of EG, act as separators between layers, migrating towards the graphite sheets and opening the graphite layers. This forms EG while maintaining the overall layered structure of the GF. As a result, the EG layer spacing varies, leading to a noticeably higher 2θ angle than rGO (2θ = 25.0°, d-spacing of ∼3.56 Å), as indexed by Feng, H et al. [49]. Furthermore, the absence of the GO diffraction peak (001) at 2θ = 11º confirms that graphite sheets have been exfoliated [50].

For Ag@EG composite catalysts, the crystal structure of EG is maintained. Additional diffraction peaks at 2θ = 37.9º, 44.2º, 64.2º and 77.2º correlate to the face-centered cubic crystal structure of AgNPs with miller indices (111), (200), (220), and (311), respectively [51]. These reflections match the AgNPs standard card (JCPDS card no. 04-0783, space group Fm-3 m (225)). These additional peaks indicate intimate integration between AgNPs and EG sheets through the formation of Ag@EG composite catalysts. Regarding the reported data [52, 53], the absence of other silver oxides (AgO_x_) in the XRD spectrum validates the complete salt-to-metal conversion and the purity of the synthesized Ag nanoparticles.

Table 1 depicts no discernible difference in the spacing between the graphitic basal planes of d_002_ and d_004_ across the three samples, suggesting that the layered structure of the EG is unaffected by metallic silver modification. In contrast, the (002) and (004) diffraction signals of Ag@EG(0.5:1) and Ag@EG(1:1) are much weaker and exhibit a decrease in full width at half maximum (FWHM). AgNPs obscure the EG diffracted beam, indicating that EG undergoes structural modifications (positional disorder) in composite samples without destroying the graphite crystal structure. This is attributed to metallic silver intercalation within the graphite layers, accompanied by an increase in crystallite size [54, 55]. From XRD data (Table 1), Scherrer’s formula (Eq. 4) is adopted to estimate the average crystallite size (Lc) of the prepared materials.

4 $$\:\text{L}\text{c}\:=\frac{\text{K}.{\uplambda\:}}{{\upbeta\:}.\:\text{c}\text{o}\text{s}{\uptheta\:}}$$

Where β is the full-width at half-maximum (FWHM) of the diffraction peaks, and K is the crystallite shape factor (assumed to be 0.9). At the diffraction angle of 26.4º, the computed crystallite size (Lc) for EG is ~ 26.6 nm, while it is larger for the composite samples Ag@EG(0.5:1) and Ag@EG(1:1), which are 53.3 and 46 nm, respectively. This confirms that the composite of EG layers and AgNPs has been effectively incorporated.

#### Raman spectroscopy analysis

**Fig. 4:**
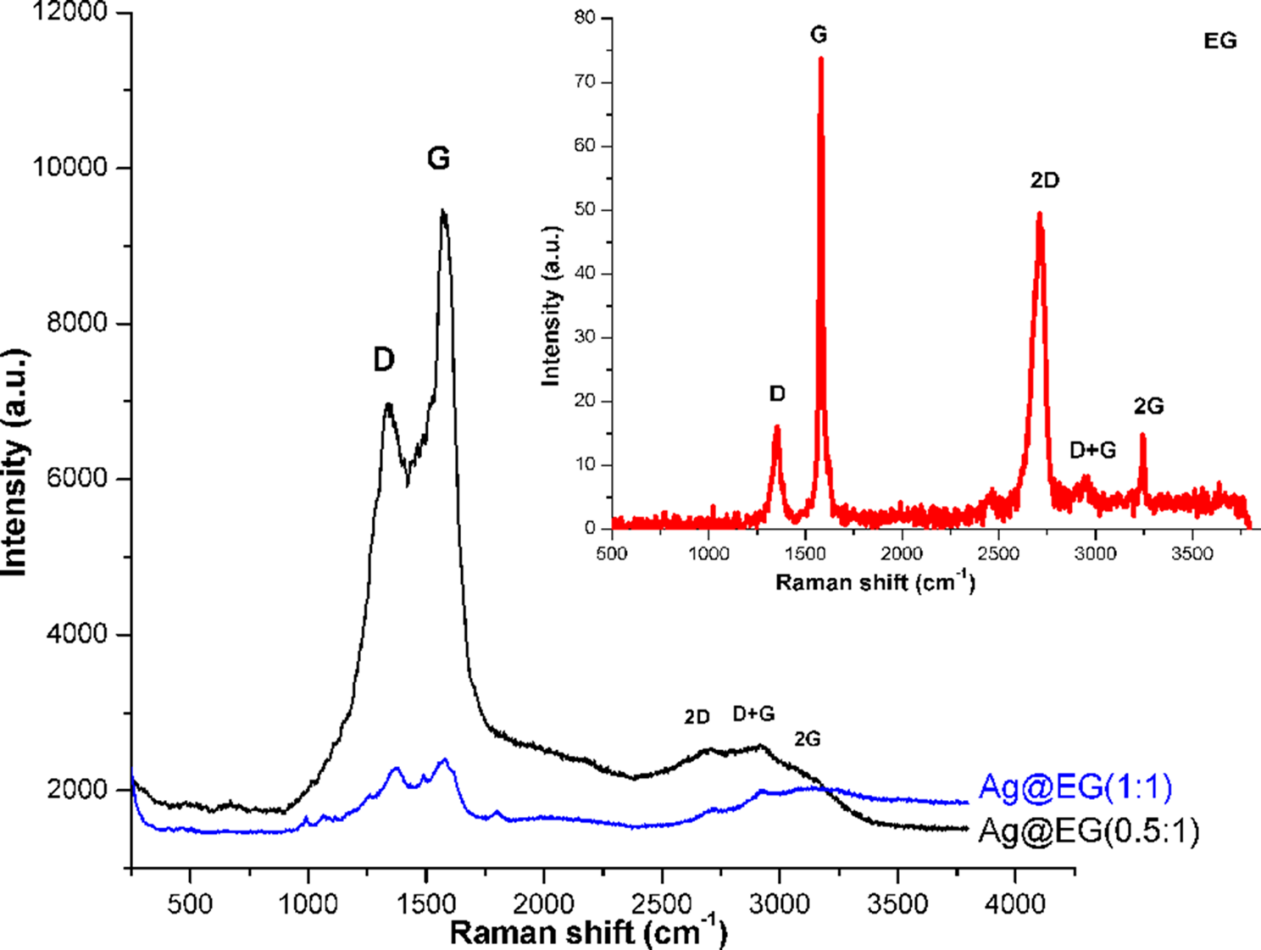
Raman spectra of as-synthesized catalysts

Raman spectroscopy is a valuable tool for characterizing graphene-based materials and validating structural disorder induced in graphite during composite formation [56, 57]. The Raman data are listed in Table 1, while Fig. 4 displays the Raman spectra of the EG and the Ag@EG composites. The spectra of all the samples show two conspicuous peaks, distinctive of native graphite, around the D and G bands, with differences in position and relative intensity. The Raman spectrum of native EG illustrates a D band at 1355 cm^− 1^ and a G band at 1580 cm^− 1^ in the first-order region. The ordered graphite (or sp^2^ carbon) is associated with the G-band, while disordered graphite (or sp^3^ carbon) is responsible for the D-band. The D-band is assigned to the in-plane carbon ring breathing mode with A_1g_ symmetry, activated by defects. The G-band reflects the E_2g_ mode, linked to sp^2^ carbon atom vibration within the two-dimensional hexagonal lattice [58, 59]. Furthermore, several bands appear in the second-order area spanning from 2500 to 3400 cm^− 1^, namely the 2D, D + G, and 2G bands at 2718 cm^− 1^, 2950 cm^− 1^, and 3245 cm^− 1^, respectively.

All bands shifted to lower wavenumbers with wider peaks due to the intercalation of AgNPs into the EG sheets. Consequently, the D and G bands’ Raman intensities increased, raising the I_D_/I_G_ intensity ratio (Table 1). The I_D_/I_G_ ratio increased from 0.22 for EG to 0.74 and 0.95 for Ag@EG (0.5:1) and Ag@EG(1:1), respectively. The increase in the I_D_/I_G_ ratio is attributed to the insertion of AgNPs into EG sheets [60]. As the surface-enhanced Raman scattering (SERS) from the forceful local electromagnetic fields of AgNPs, accompanied by Plasmon resonance, accounts for this rise [61]. This, in turn, leads to the structural disordering of the EG matrix in the composite catalysts [62]. This structural disorder could be ascribed to the in situ development of AgNPs into EG sheets, weakening the bonding of sp^2^-hybridized C atoms, and the close contact between AgNPs and the carbon matrix, raising the EG defect level. The matching between this result and the corresponding PXRD measurement accords rather well.

**Table 1:** Crystallographic parameters and Raman shift positions with the intensity ratio (I_D_/I_G_) of prepared catalysts

Characteristic assignments	EG	Ag@EG(0.5:1)		Ag@EG(1:1)	
**Crystallographic parameters**					
*d*_*002*_/nm	0.337		0.3369		0.3364
*d*_*004*_/nm	0.1683		0.1683		0.1682
Lc_(002)_/nm	26.6		53.3		46
**Raman scattering parameters**					
D/cm^− 1^	1355		1349		1353
G/cm^− 1^	1580		1573		1567
2D/cm^− 1^	2718		2704		2711
D + G/cm^− 1^	2950		2915		2922
2G/cm^− 1^	3245		3175		3132
I_D_/I_G_	0.22		0.74		0.95

#### FT-IR and UV-Vis spectroscopic analyses

**Fig. 5:**
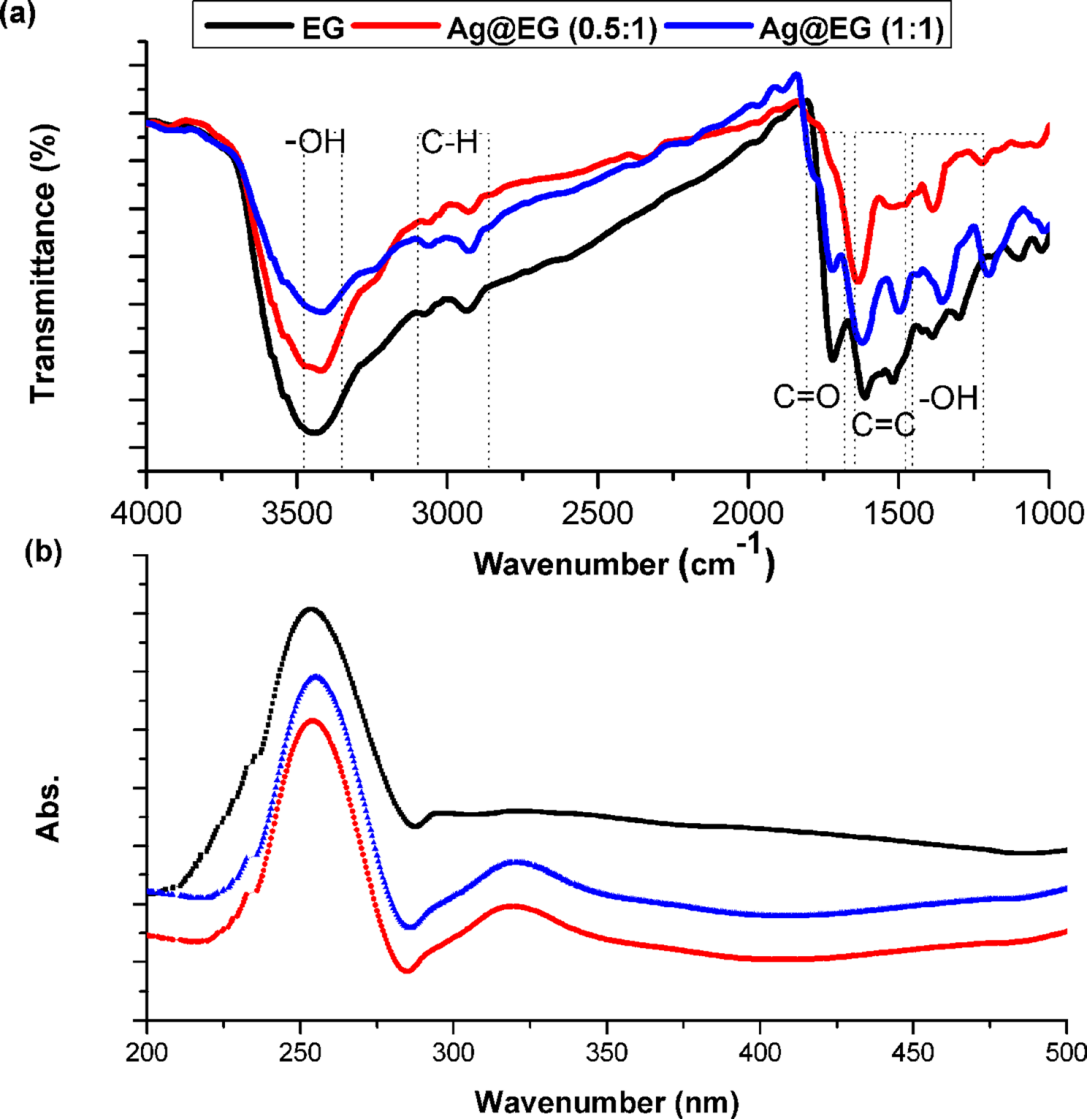
(**a**) FTIR and (**b**) UV-Vis. spectra of fabricated catalysts

FT-IR analysis was accomplished to detect the structural amendments of the prepared catalysts. As considered in Fig. 5(a), each specimen demonstrated broad and strong absorption peaks in the 3400–3000 cm^-1^ and 1380 cm^-1^ ranges [63], corresponding to the anti-symmetry and stretching vibration of hydroxyl in free water. The sp^2^-hybridized C=C of EG was appointed by the asymmetrical stretching vibration peak at 1610 cm^-1^ [64]. The absorption peak at approximately 1700 cm^-1^ represented the stretching vibrations of C = O functional groups [65]. The incorporation of APS through the process created EG with a remarkably low degree of oxidation, as evidenced by the detection of OH and C = O functional groups on the EG surface. No other functional groups with oxidation characteristics were recognized [39]. The substantial decrease in the intensity of the oxygenated functional groups of the synthesized Ag@EG composite catalysts with the two ratios is attributed to the presence of AgNPs on the EG surface. Table 2 reflects a slight shift in a few peaks throughout the incubation of AgNPs, revealing that the oxygen moieties of EG interact electrostatically with Ag [66]. Conversely, the other distinctive peaks of EG remaining in the same position imply no change or reaction in these parts when silver was integrated.

Figure 5(b) displays the UV-Vis spectra of the EG and Ag@EG composite catalysts, verifying the development of AgNPs. For EG, the electronic π→π* transitions of aromatic C-C bonds are responsible for the largest absorption peak at 250 nm, while C = O bonds are highlighted by the moderate shoulder at approximately 300 nm [50]. The absorption spectra of Ag@EG exhibit no shift in the maximum peak of EG at 250 nm; rather, it remains at the same location but with less intensity. The characteristic surface plasmon resonance (SPR) band of AgNPs, initially observed at approximately 400 nm, shifted to a shorter wavelength of 320 nm in the present study. This indicates a shift to a lower pH value, consistent with the work reported by P.Traiwatcharanon et al. [67].

**Table 2:** FTIR and UV-Vis. Characteristic band assignments of prepared catalysts

Band assignments	Frequency (cm^− 1^)		
	EG	Ag@EG (0.5:1)	Ag@EG (1:1)
-OH	3432 1380	3428 1382	3422 1355
C-H	3073 2935	3066 2925	3062 2927
C = O	1722	1714	1718
C = C	1610	1633	1617
**Frequency (nm)**			
π → π * of aromatic C-C	250	250	250
C = O	300	300	300
Plasmon resonance (SPR)	-	320	320

### BET-measurements

**Fig. 6:**
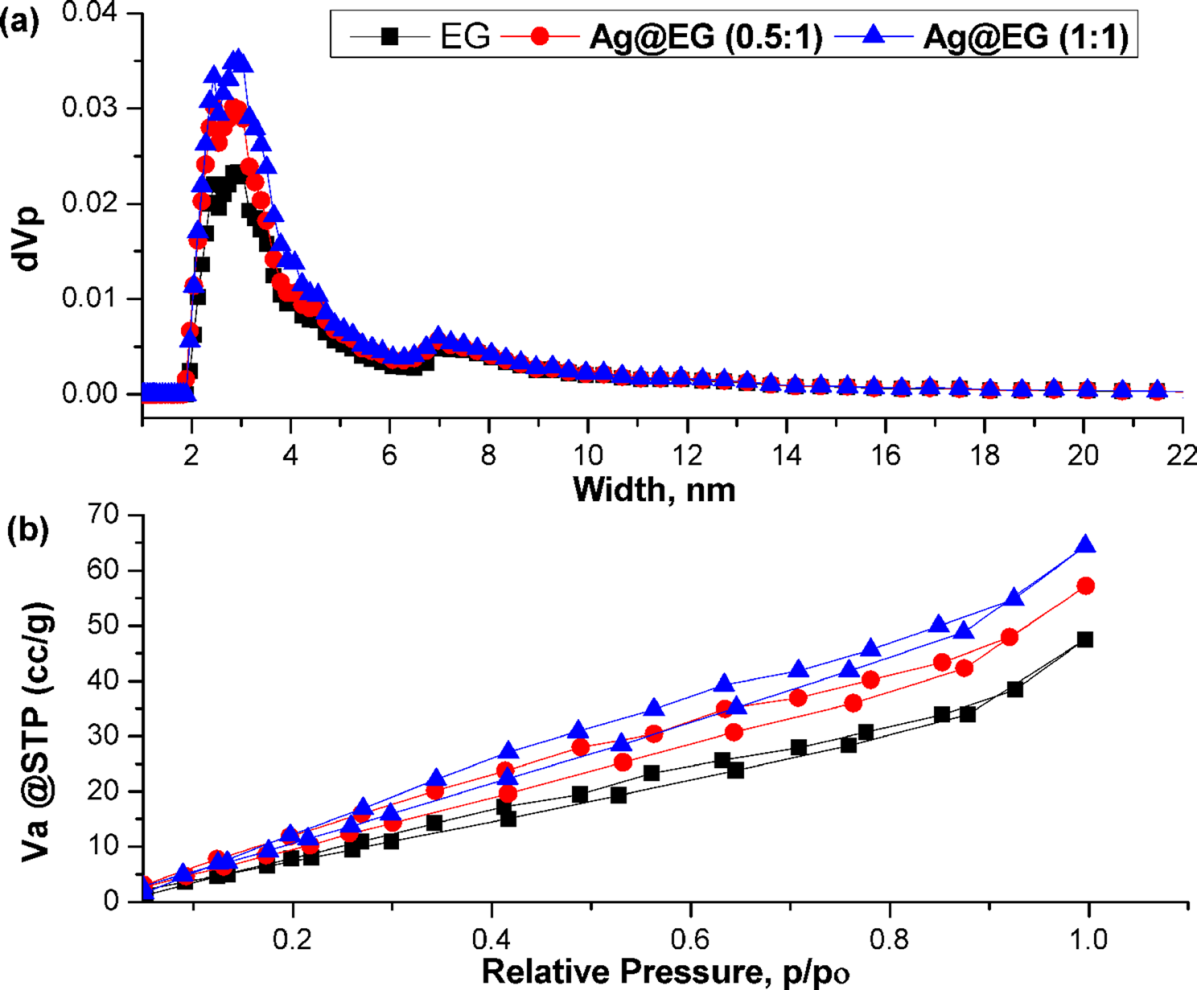
(**a**) pore size distribution, and (**b**) N_2_ adsorption–desorption isotherm curves of prepared catalysts

Figure 6(a, b) illustrates the pore-size distributions (PSD) and nitrogen adsorption–desorption isotherms, respectively, for the EG, Ag@EG (0.5:1), and Ag@EG (1:1) composite catalysts. Analysis of Fig. 6(a) indicates that all three samples exhibit uniform mesoporous characteristics, with prominent pore distribution peaks at 2.4, 3, and 7 nm. As shown in Table S1, the BET-surface area (S_BET_) of the prepared samples increased with higher AgNPs concentration, following the order: EG > Ag@EG (0.5:1) > Ag@EG (1:1). This indicates that adding AgNPs significantly impacts the surface properties of the resulting composite catalysts. Additionally, the Ag@EG composites possess a larger total pore volume than the EG material. All three samples display Type IV isotherms with H1 hysteresis loops (according to IUPAC classification [68]), indicating a porous structure (Fig. 6b). As AgNPs concentration increases, the adsorbed volume clearly rises, suggesting enhanced porosity [69]. This improvement in total pore volume, S_BET_, and mesoporous characteristics due to Ag incorporation directly correlates with an increase in active functional sites on the composite. This leads to enhanced removal capacity, as confirmed by the improved degradation activity of the composite catalysts towards MB dye.

### Fenton-like catalytic degradation of MB dye

**Fig. 7:**
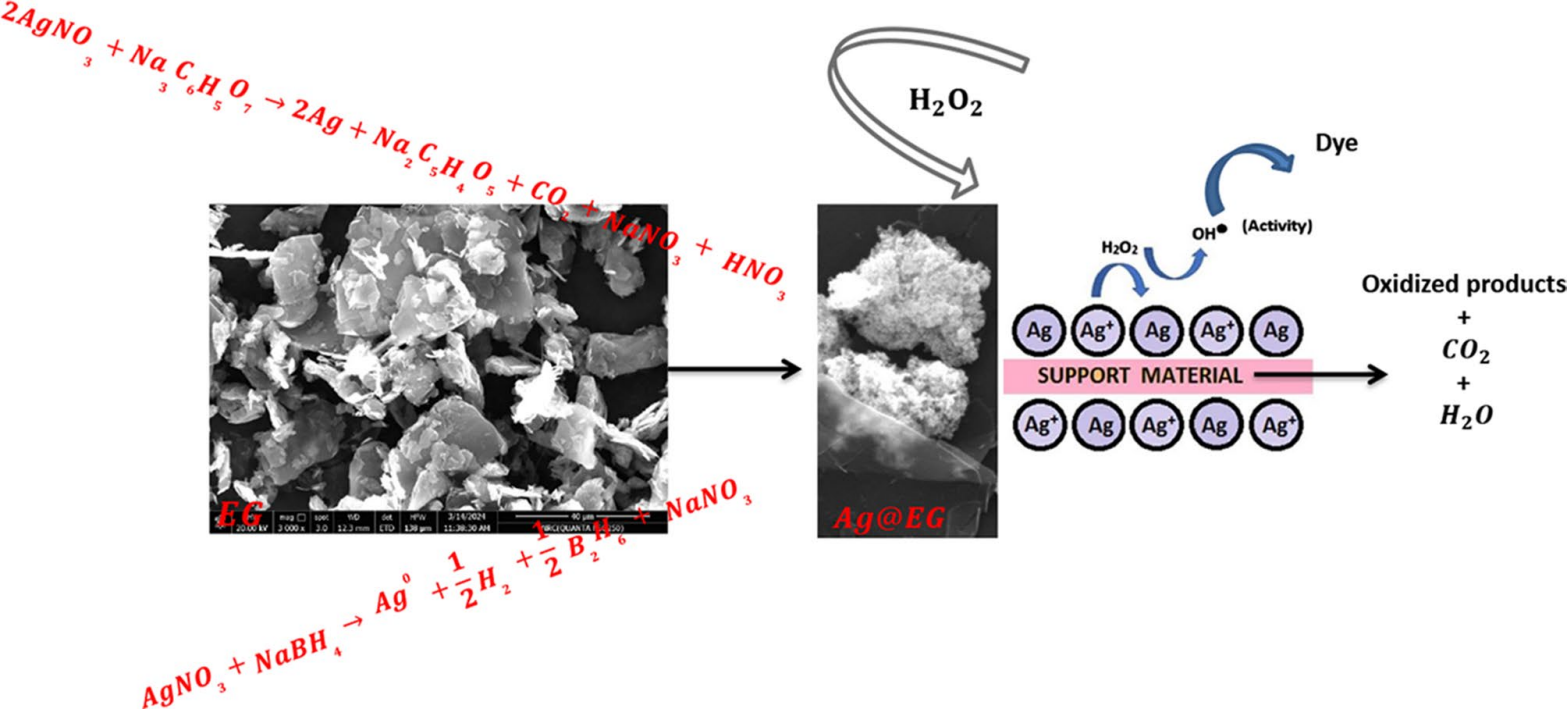
Schematic representation for decorating Ag@EG and its catalytic oxidation of MB dye

By exploiting the Fenton mechanism, silver-based materials can serve as catalysts to degrade organic contaminants in the presence of H_2_O_2_. When organic pollutants comprise heteroatoms, like MB dye, the hydroxide or peroxide radicals generated during silver oxidation can initiate the breakdown of these pollutants into carbon dioxide, water, and inorganic salts [70] (Fig. 7). In the current system, the concentrations of [H_2_O_2_], [MB dye], pH, reaction duration, and temperature (298–318 K) were optimized.

#### Effect of pH

In Fenton-like oxidation, silver can exist in two oxidation states (Ag^0^ or Ag^+^), depending on the pH of the aqueous medium. Therefore, pH is a particularly essential variable [71]. In acidic environments, bare silver tends to dissolve in the aqueous phase and catalytically convert H_2_O_2_ into HO• radicals through a homogeneous reaction (Eq. 5).

5 $$\:\text{A}\text{g}+{\text{H}}_{2}{\text{O}}_{2}\to\:{\text{A}\text{g}}^{+}\:+\:{\text{O}\text{H}}^{-}+\:{\text{H}\text{O}}^{{\bullet\:}}$$

Nevertheless, basic settings will cause the redox reaction to change in the opposite direction, producing O_2_ through a heterogeneous phase reaction instead of the development of HO^•^ radicals (Eq. 6) [72].

6 $$\:{\text{A}\text{g}}^{+}\:+\:{\text{O}\text{H}}^{-}+\:\text{H}\text{O}{}_{2}^{{\bullet\:}}\:\:\to\:\text{A}\text{g}+{\text{O}}_{2}+{\text{H}}_{2}\text{O}$$

**Fig. 8:**
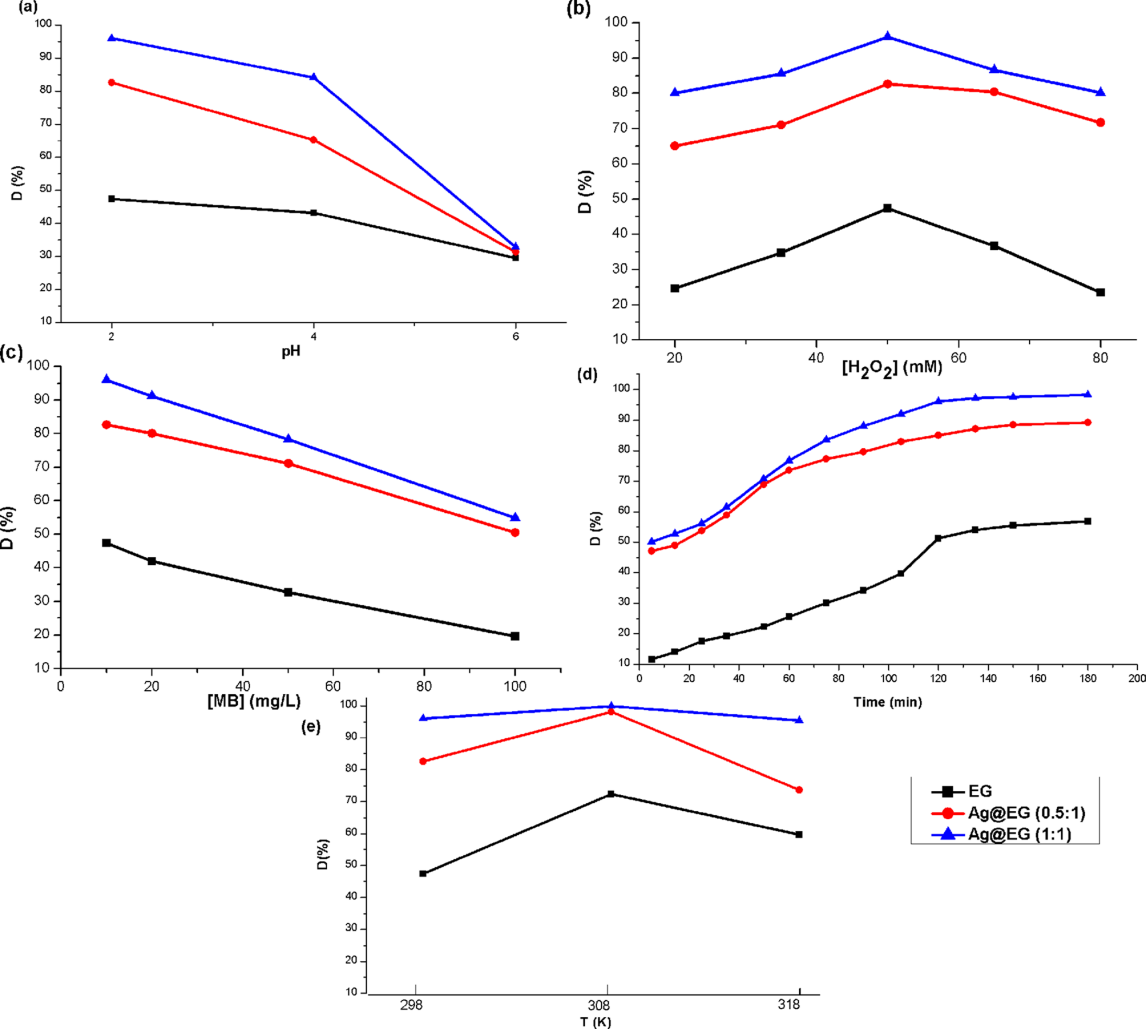
Effect of Fenton operating conditions; (**a**) pH, (**b**) oxidant concentrations, (**c**) dye concentrations, (**d**) time, and (**e**) temperature on the decolorization efficiency of MB dye using prepared catalysts

Accordingly, the consequence of pH on MB degradation was inspected at pH 2.0, 4.0, and 6.0, while holding the oxidant and dye concentrations at 50 mM and 10 mg.L^− 1^, adequately, at room temperature. As shown in Fig. 8(a), under these conditions, the MB degradation rate significantly decreased with the increment of pH. At pH 2, MB was almost completely degraded within 120 min, with decolorization percentages of 47%, 83%, and 96% for EG, Ag@EG (0.5:1), and Ag@EG (1:1), respectively. However, the MB degradation percentages reached 43%, 65.3%, and 84% at pH 4, and 29.5%, 31.3%, and 33% at pH 6 for EG, Ag@EG (0.5:1), and Ag@EG (1:1), respectively, after 120 min. This reflects that a lower pH enhanced the reaction between Ag^0^ and H_2_O_2_, generating HO^•^, which resulted in a stronger oxidation ability [73].

#### Effect of oxidant concentration

To gauge the optimum [H_2_O_2_], different concentrations of H_2_O_2_ (20–80 mM) were conducted at a dye concentration of 10 mg.L^− 1^, maintaining the solution’s pH at 2 and the temperature at 298 K for 120 min. A correlation exists between the degradation efficiency of the dye and H_2_O_2_ concentrations in the Fenton process, as shown in Fig. 8(b). As [H_2_O_2_] increased from 20 mM to 50 mM, the decolorizing rate of MB dye elevated, achieving maximum dye degradation (47%, 83%, and 96%) for EG, Ag@EG (0.5:1), and Ag@EG (1:1), adequately. However, when [H_2_O_2_] increased above 50 mM, the decolorization efficiency dropped. The detected variation in the dye’s degradation might be attributed to the scavenging of HO^•^ at high H_2_O_2_ concentrations, which leads to the creation of a perhydroxyl radical, HO_2_^•^, that is less reactive than HO^•^ [74], as shown in Eq. 8. Furthermore, it could keep reacting with hydroxyl radicals to produce O_2_ (Eq. 8), which consumes some hydroxyl radicals and prevents MB from mineralizing.

7 $$\:{\text{H}}_{2}{\text{O}}_{2}+{\text{H}\text{O}}^{{\bullet\:}}\:\:\to\:\:{\text{H}}_{2}\text{O}+\:{\text{H}\text{O}}_{2}^{{\bullet\:}}$$

8 $$\:{\text{H}\text{O}}_{2}^{{\bullet\:}}\:+{\:\text{H}\text{O}}^{{\bullet\:}}\:\:\to\:\:\text{H}{}_{2}\text{O}+{\text{O}}_{2}$$

An optimal H_2_O_2_ dose of 50 mM for the removal of MB dye was documented by Saeedah et al., who declared a degradation efficiency of 95.5% for crystal violet dye [75].

#### Effect of dye concentration

Since the organic concentration of wastewater varies, research was conducted on the impact of the initial dye concentration. The findings are highlighted in Fig. 8(c), with an initial concentration range of 10–100 mg.L^− 1^ and implementing conditions (pH. 2, [H_2_O_2_]. 50 mM, contact time. 120 min, and T. 298 K). As shown in Fig. 8(c), increasing the initial concentration of MB from 10 to 100 mg.L^− 1^ dramatically decreased the color degradation from (47, 83, and 96%) to (19.5, 50.5, and 55%) for EG, Ag@EG (0.5:1), and Ag@EG (1:1), respectively. The dye molecules may have blocked the catalyst’s silver sites at high concentrations, decreasing the material’s catalytic activity. Consequently, the development of hydroxyl radicals was impeded [76]. Finally, an initial concentration of 10 mg.L^− 1^ was appointed to administrate the degradation experiments.

#### Effect of contact time and temperature

The influence of reaction time and temperature on the decolorization performance of MB dye was examined and demonstrated in Fig. 8(d, e), respectively, as they are crucial factors for oxidation reactions. The degradation efficacy of the MB dye started after 5 min., achieving D% (11.5, 47, and 50%) for EG, Ag@EG (0.5:1), and Ag@EG (1:1), respectively. The decolorization rate increased smoothly till 90 min, reaching equilibrium and maximum removal efficiencies of 47% for EG, 83% for Ag@EG (0.5:1), and 96% for Ag@EG (1:1) after 120 min of treatment.

Most commercial applications of the Fenton reagent occur at temperatures between 298 and 318 K. Therefore, the consequence of temperature on Fenton treatment within this range was probed through Fig. 8(e). Raising the temperature positively impacts the decolorization of MB dye. The decolorization efficiency increased to 72.4, 98.1, and 99.8% for EG, Ag@EG (0.5:1), and Ag@EG (1:1), respectively, at 298, 308, and 318 K after 90 min of reaction time. This results from the high temperature speeding up the catalyst-hydrogen peroxide interaction, which in turn accelerates the production of oxidizing species like HO^•^ radicals and high-valence silver species [77]. Additionally, reactant molecules may have more energy at a higher temperature to overcome the reaction activation energy [78].

Table 3 reveals a comparative study for the catalytic degradation of MB dye under optimum conditions. The decolorization efficiency of MB dye is greatly enhanced under optimal operating conditions (pH.2, [H_2_O_2_].50 mM, [MB].10 mg.L^− 1^, T. 318 K, and time.120 min) applying a Fenton-like process with the prepared composite catalyst, achieving the highest D% (99.8%) for Ag@Eg (1:1).

**Table 3:** Comparative study of catalytic degradation of MB dye using our prepared catalysts regarding to other prepared samples

Catalyst	D (%)	Optimum operating conditions				Reference
		pH	[H_2_O_2_] (mM)	Time (h)	T (K)	
Mn/Ti-HMS	63.9	7	10	2	298	[79]
EG	72.4	2	50	2	318	This study
Fe^II^@MIL-100(Fe)	91	3	40	5	298	[80]
Fe_2_O_3_ Over CeO_2_	94	9.6	640	7	300	[72]
CuFe-MC-1–800	95.4	3	30	1	298	[81]
Fe_0_–Fe_3_O_4_–RGO	98	3	0.8	1	298	[82]
Ag@EG (0.5:1)	98.1	2	50	2	318	This study
Ag@EG (1:1)	99.8	2	50	2	318	This study
CuO over CeO_2_	99	9.6	640	7	300	[72]
Fe_3_O_4_ Nanoparticle	100	3	1	0.5	298	[83]
Fe_0_/Fe_3_O_4_	100	6	300	2	303	[84]

### Mechanism of Fenton-like catalytic degradation of MB dye

The process of dye degradation can be better understood by examining the effects of temperature and time on Fenton-like oxidation. To establish the kinetics of MB dye degradation on produced materials using the Fenton reaction, pseudo-first-order and pseudo-second-order kinetic models were evaluated. The general elementary rate law for the MB dye reaction may be formulated as follows:

9 $$\:\text{r}=-\frac{\text{d}{\text{C}}_{\text{M}\text{B}}}{\text{d}\text{t}}={\text{K}}_{\text{O}\text{H}}{\text{C}}_{\text{H}\text{O}{\bullet\:}}{\text{C}}_{{\text{M}\text{B}}_{^\circ\:}}+{\sum\:}_{\text{i}}^{}{\text{K}}_{\text{o}\text{x}\text{i}}{\text{C}}_{\text{o}\text{x}\text{i}}{\text{C}}_{\text{M}\text{B}}$$

Other oxidants, such as the perhydroxyl radical HO_2_•, are represented by the term Oxi. The HO• can be considered the sole active oxidant for simplification. Direct measurements of HO• concentration are not possible. Certain reaction conditions contribute to a pseudo-first-order reaction, where the HO• concentration is included in the apparent rate constant, K_app_. Under these conditions, [HO•] is constant [75].

10 $$\:\text{r}=-\frac{\text{d}{\text{C}}_{\text{M}\text{B}\:}}{\text{d}\text{t}}={\text{K}}_{\text{a}\text{p}\text{p}}{.\:\text{C}}_{{\text{M}\text{B}}_{^\circ\:}}$$

By integration, the rate law of pseudo-first-order reaction can be formulated as follows:

11 $$\:{\:\text{l}\text{n}\text{C}}_{\text{M}\text{B}}=\text{ln}{{\text{C}}_{\text{M}\text{B}}}_{^\circ\:}-{\text{K}}_{1}.\text{t}$$

where C_MB°_ symbolizes the initial concentration of MB dye and can be ascertained using the initial dye absorbance (A_°_). C_MB_ is the concentration of MB dye at time t and can be revealed using dye absorbance at time (A_t_). Consequently, Eq. 11 can be modulated to:

12 $$\:\text{ln}\left(\frac{{\text{A}}_{\text{t}}}{{\text{A}}_{^\circ\:}}\right)=\:-{\text{K}}_{1}.\:\text{t}$$

A straight line with a negative slope was produced when $\:\text{ln}\left(\frac{{\text{A}}_{\text{t}}}{{\text{A}}_{^\circ\:}}\right)\:$was plotted against time. The K_1_ value for the disintegration of the organic target compound is denoted by the slope of this line. The pseudo-second-order reaction’s rate law may be expressed as follows:

13 $$\:\frac{1}{\left[{\text{A}}_{\text{t}}\right]}={\text{K}}_{2}.\text{t}+\frac{1}{\left[{\text{A}}_{^\circ\:}\right]\:}$$

**Fig. 9:**
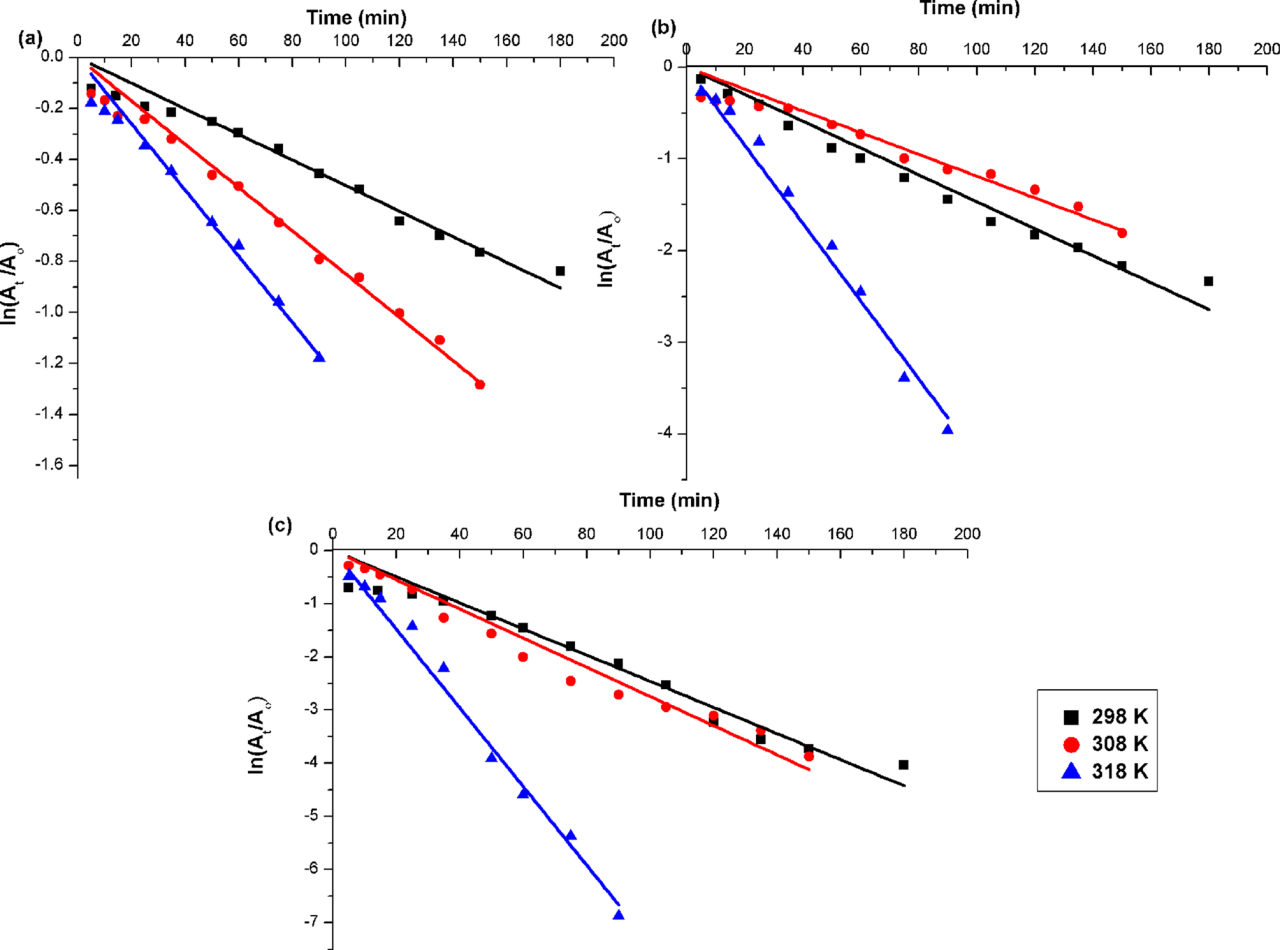
Pseudo-first-order kinetic study for Fenton-like oxidative degradation of MB dye (20 mg.L^− 1^) by; (**a**) EG, (**b**) Ag@EG (0.5:1), and (**c**) Ag@EG (1:1) at T. 298–318 K, pH. 2, [H_2_O_2_]. 50 mM

K_1_ and K_2_ represent the apparent rate constants of the pseudo-first-order (min^− 1^) and pseudo-second-order equations (min^− 1^). The consequences of both equations are displayed in Figs. 9 and S3, respectively. Calculated rate constants and correlation coefficients (R^2^), are outlined in Table 4. According to Table 4, the R^2^ values for the pseudo-first-order equation are higher than those for the pseudo-second-order equation. The K_1_ rate constant increases with temperature: from 0.005 to 0.013 for EG, from 0.015 to 0.043 for Ag@EG (0.5:1), and from 0.025 to 0.074 for Ag@EG (1:1). The Fenton degradation process of MB is better elucidated by the pseudo-first-order kinetic model.

**Table 4:** Kinetic constants of catalytic degradation for MB dye employing as-prepared catalysts

Parameters		T (K)	EG	Ag@EG (0.5:1)	Ag@EG (1:1)
Pseudo-first order	K_1_(min^− 1^)	298 308 318	0.005 0.0085 0.013	0.015 0.012 0.043	0.025 0.027 0.074
	R^2^	298 308 318	0.9899 0.9943 0.9924	0.9914 0.9809 0.9941	0.9876 0.9886 0.9952
Pseudo-second order	K_2_(min^− 1^)	298 308 318	0.003 0.005 0.01	0.023 0.009 0.18	0.1 0.076 0.18
	R^2^	298 308 318	0.8831 0.8186 0.884	0.893 0.8856 0.7431	0.839 0.8253 0.793

### Thermodynamic studies

As stated, the apparent first-order kinetic rate constants at different temperatures (298, 308, and 318 K), activation energy (Ea), and some thermodynamic parameters were estimated based on the Arrhenius Eq. [4]:

14 $$\:{\text{K}}_{\text{a}\text{p}\text{p}}=\text{A}.\text{exp}\left(-\frac{{\text{E}}_{\text{a}}}{\text{R}.\text{T}}\right)\:\:$$

where A is a pre-exponential factor (or frequency; min^− 1^), Ea is the apparent activation energy (J.mol^− 1^), R is the ideal gas constant (8.314 J.mol^− 1^.K^− 1^), T is the absolute temperature (K), and K_app_ denotes the apparent first-order rate constant (min^− 1^). Equation (14) can be declared in its linearized form as Eq. 15:

15 $$\:\text{l}\text{n}{\text{K}}_{\text{a}\text{p}\text{p}}=\:-\frac{{\text{E}}_{\text{a}}}{\text{R}\text{T}}+\text{l}\text{n}\text{A}$$

**Fig. 10:**
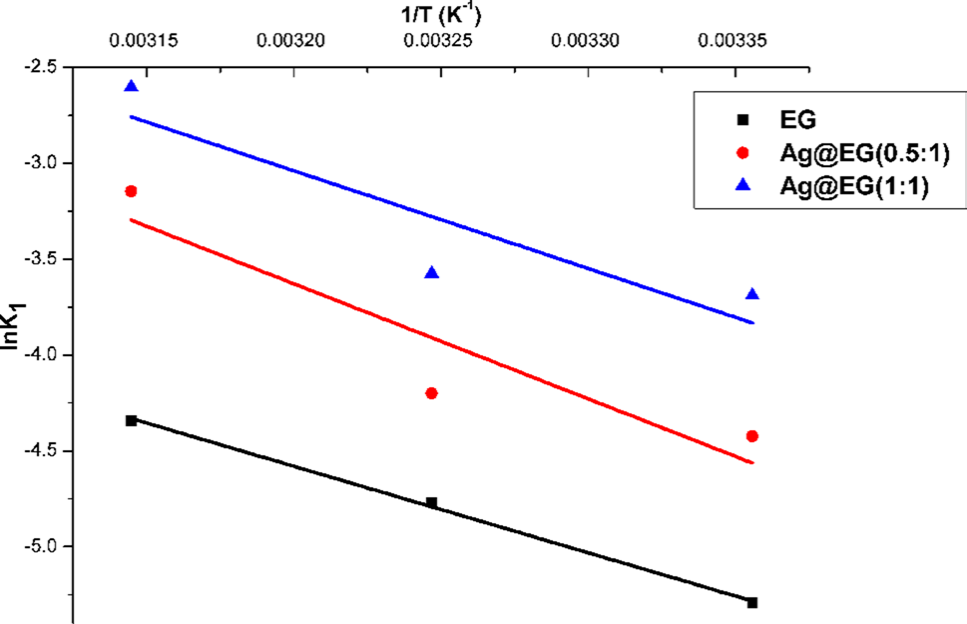
Arrhenius plot of Fenton-like catalytic degradation for MB dye

The Arrhenius plot of lnK versus 1/T authorized assessment of apparent activation energy values from the straight-line slopes of Fig. 10. The attained findings are detailed in Table 5. Activation energy represents the minimum energy that reactants must possess for the reaction to proceed. The activation energy values were moderate, at 37.5, 49.9, and 42.4 kJ/mol for EG, Ag@EG (0.5:1), and Ag@EG (1:1), adequately. These values imply that the oxidative reaction proceeded with a low energy barrier.

The quantity of activation energy demonstrates the type of removal, which might be chemical or physical. The magnitude of Ea ranging from 5 to 40 kJ/mol, corresponding to a physisorption mechanism, is observed for EG. However, a higher range of values (40–800 kJ/mol) observed for Ag@EG (0.5:1) and Ag@EG (1:1) suggests that a chemisorption mechanism can be ruled out [4].

Catalysts work by lowering the activation energy of a reaction, thereby increasing the reaction rate. Hence, kinetic and thermodynamic parameters reveal that Ag@EG (1:1) has lower activation energy values and a higher kinetic rate constant than Ag@EG (0.5:1), confirming silver’s catalytic efficiency for dye degradation. Table 5 depicts a lower comparative activation energy for our prepared catalysts compared to other prepared samples.

The Erying-Polanyi equation was applied to determine the thermodynamic aspects, taking into account data derived from Fig. 10.

16 $$\:\text{ln}\left(\frac{\text{K}}{\text{T}}\right)=\:-\frac{{\Delta\:}{\text{H}}^{\text{*}}\:}{\text{R}}.\frac{1}{\text{T}}+\text{ln}\left(\frac{{\text{K}}_{\text{B}}}{\text{h}}\right)+\frac{{\Delta\:}{\text{S}}^{\text{*}}}{\text{R}}$$

**Fig. 11:**
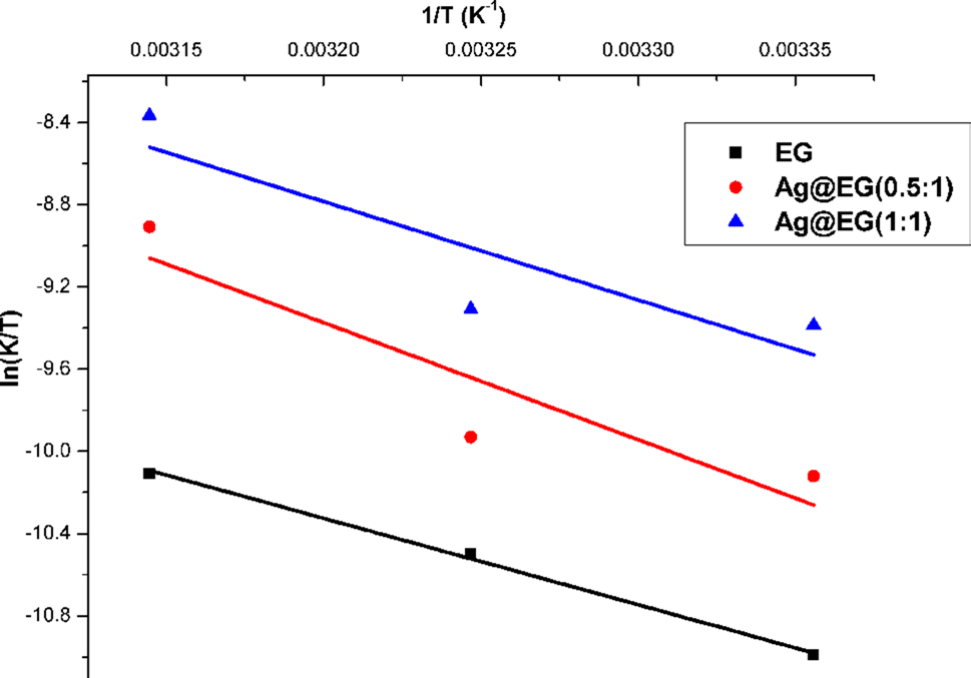
Thermodynamic studies of Fenton-like catalytic degradation for MB dye

Where k_B_ presents the Boltzmann constant (1.3806 × 10^− 23^ m^2^.kg.min^− 2^.K^− 1^), ΔH* and ΔS* serve as the enthalpy (kJ/mol) and entropy (J/mol.K), respectively, K is the apparent rate constant (min^− 1^), and h implies the Blank constant (6.626 × 10^–34^ m^2^.kg/min). The plot of ln (K/T) vs. 1/T in Fig. 11 designates a straight line, and the values of ΔS* and ΔH* can be acquired from the intercept and slope, respectively. The value of ΔG*, Gibbs free energy (kJ/mol), can be computed from the stated equation [75]:

17 $$\:{\Delta\:}{\text{G}}^{\text{*}}\:=\:{\Delta\:}{\text{H}}^{\text{*}}-\text{T}.{\Delta\:}{\text{S}}^{\text{*}}$$

The thermodynamic parameters are evidenced in Table 5. The negative ΔS* values (-171.2, -124, and − 143 J/mol.K) for EG, Ag@EG (0.5:1), and Ag@EG (1:1), respectively, indicate a reduction in randomness and degrees of freedom within the system where degradation occurred. These findings suggest that the as-prepared catalysts have an energetically favorable impact, proving their effectiveness [85]. Considering the retention of positive ΔG* values, the oxidative reaction involving the Fenton reagent for MB degradation is proposed to be non-spontaneous [4]. The MB degradation is endothermic due to the positive ΔH* values (35, 47.4, and 39.4 kJ/mol for EG, Ag@EG (0.5:1), and Ag@EG (1:1), respectively).

**Table 5:** Thermodynamic constants of catalytic degradation for MB dye using as-prepared catalysts and other indexed samples

Thermodynamic parameters	T (K)	E_a_ (k.J/mol)	ΔH* (k.J/mol)	ΔS* (J/mol.K)	ΔG* (k.J/mol)	References
EG	298	37.5	35	-171.5	86.3	**This study**
	308				88	
	318				89.7	
Ag@EG (0.5:1)	298	49.9	47.4	-124	84.4	**This study**
	308				85.6	
	318				87	
Ag@EG (1:1)	298	42.4	39.4	-143	82	**This study**
	308				83.4	
	318				85	
Sulphuric acid-treated activated carbon (SAC)	303	29.7	29.8	129.2	-9.51	[4]
QAMPSA/VPFe_3_O_4_	303	88.93	86.30	0.665	-	[70]
Syrian natural magnetite	303	18.02	15.38	-219.8	-51.24	[86]
Ferrous catalyst (FeSO_4_)	298.15	31.5	29	196	29.4	[87]
Haematite nanocrystals	313	79.01	48.67	139.31	92.27	[88]

## Conclusion

Catalytic degradation of water pollutants can be assessed as an economical and energy-efficient procedure for wastewater treatment. In this work, new silver-based composite catalysts were prepared using a mixture of Na_3_C_6_H_5_O_7_ and NaBH_4_ as reducing agents for AgNO_3_ to in situ decorate exfoliated graphite (EG) with AgNPs in two ratios (Ag@EG (0.5:1) and Ag@EG (1:1)). XRD and SEM analyses verified the attachment of connected metal framework clusters from AgNPs on the EG surface with a diameter range of 45 ± 13 nm, and 15% and 35% Ag content for Ag@EG (0.5:1) and Ag@EG (1:1), respectively. Raman and UV-Vis spectra confirm the formation of characteristic surface Plasmon resonance for AgNPs. Homogeneous Fenton-like catalytic degradation of MB dye using the prepared catalysts demonstrates better decolorization efficiency under operating conditions (pH.2, [H_2_O_2_].50 mM, [MB].10 mg.L^− 1^, T. 318 K, and time.120 min), achieving the highest D% (99.8%) for Ag@EG (1:1). The kinetic correlation coefficient values (R^2^) show higher values for pseudo-first-order than for pseudo-second-order. The thermodynamic exploration illustrates moderate Ea values of 37.5, 49.9, and 42.4 kJ/mol for EG, Ag@EG (0.5:1), and Ag@EG (1:1), respectively. A reduction in randomness is indicated by the negative ΔS* values, while non-spontaneous and endothermic properties are shown by the positive ΔG* and ΔH* values, respectively.

## Electronic supplementary material

Below is the link to the electronic supplementary material.

Supplementary Material 1

Supplementary Material 2

Supplementary Material 3

Supplementary Material 4

Supplementary Material 5

## Data Availability

All data generated or analyzed during this study are included in this published article.
