# Supplementary material for: In situ decoration of Ag@exfoliated graphite composite catalyst for Fenton-like oxidation of methylene blue dye: kinetic and thermodynamic studies
Source: BMC Chem. 2025 Jul 24;19(1):221. doi: 10.1186/s13065-025-01584-1 (PMC12291263; doi:10.1186/s13065-025-01584-1)
Supplement: Supplementary file 1 — Supplementary Material 1 [file 13065_2025_1584_MOESM1_ESM.docx]

**In Situ Decoration Of Ag@Exfoliated Graphite Composite Catalyst for Fenton-Like Oxidation of Methylene Blue Dye: Kinetic and Thermodynamic Studies**

Somia M. Abbas^1*^, Khadiga M. Abas^2^*

^1^ Inorganic Chemistry Department, National Research Centre, 33 El Bohouth St., (Former El Tahrir St.), Dokki, Giza 12622, Egypt

^2^ Physical Chemistry Department, Advanced Materials Technology and Mineral Resources Research Institute, National Research Centre, 33 El-Bohouth St., Giza 12622, Egypt

^*^**Corresponding authors**: [somiamohamed223@gmail.com](mailto:somiamohamed223@gmail.com); [sa.abbas@nrc.sci.eg](mailto:sa.abbas@nrc.sci.eg) (S.M.Abbas) [[0000-0002-5192-2561](https://www.scopus.com/redirect.uri?url=https://orcid.org/0000-0002-5192-2561&authorId=57193142500&origin=AuthorProfile&orcId=0000-0002-5192-2561&category=orcidLink)]; [mohamedkhadiga728@yahoo.com](mailto:mohamedkhadiga728@yahoo.com); km.hassan@nrc.sci.eg (K.M.Abas.) **[0000-0003-2569-7484]**

Tel.: +201121121327 (S.M.Abbas); +201022197965 (K.M.Abas)

**Supplementary Information (SI)**


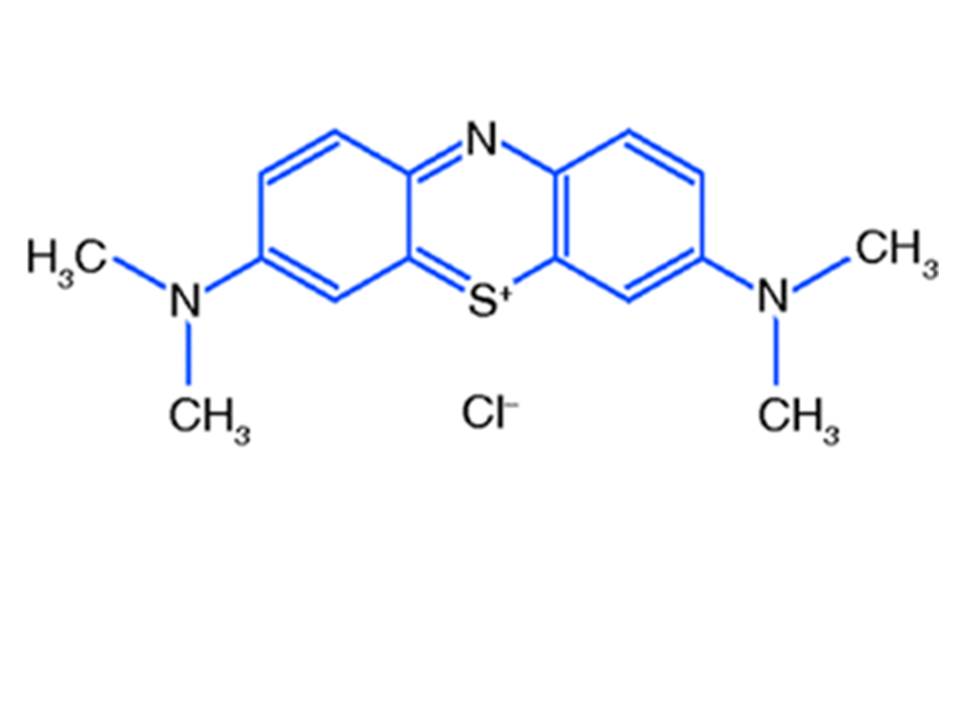


**Fig S1. Chemical Structure of MB dye.**


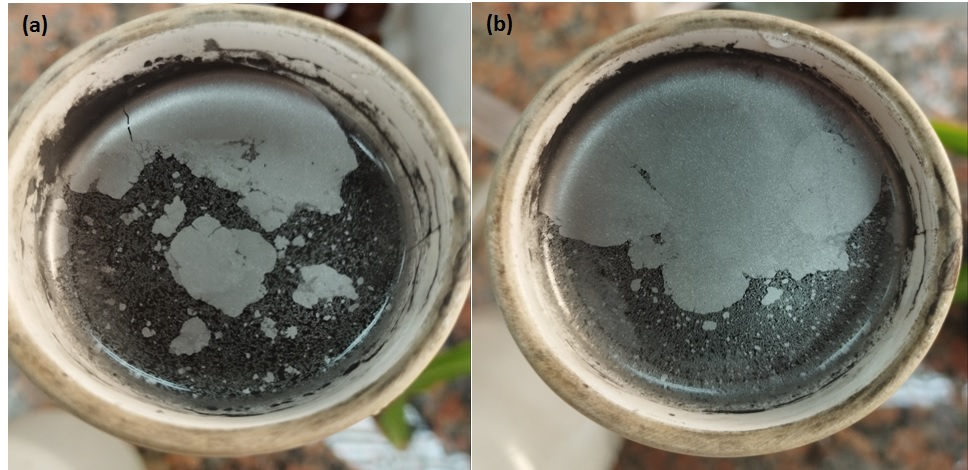


**Fig S2. Photographs representing mirror-like silver layer onto prepared composite catalysts.**

**

 Fig S3. Pseudo-second-order kinetic study for Fenton-like oxidative degradation of MB dye (20 mg/L) by; (a) EG, (b) Ag@EG (0.5:1), and (c) Ag@EG (1:1) at T. 298-318K, pH. 2, [H_2_O_2_]. 50 mM.**

| **Table S1. BET analysis of prepared catalysts.** | | | | |
| --- | --- | --- | --- | --- |
| **Ag@EG (1:1)** | **Ag@EG (0.5:1)** | **EG** | **Textural Characteristics** |  |
| 87 | 75.4 | 53.1 | **S_BET_ (m^2^/g)** |  |
| 4.6 | 4.7 | 5.5 | **Mean pore**  **diameter (nm)** |  |
| 0.1 | 0.09 | 0.07 | **Total pore**  **volume (cm^3^/g)** |  |
