# Supplementary figures and images for: In situ decoration of Ag@exfoliated graphite composite catalyst for Fenton-like oxidation of methylene blue dye: kinetic and thermodynamic studies
Source: BMC Chem. 2025 Jul 24;19(1):221. doi: 10.1186/s13065-025-01584-1 (PMC12291263; doi:10.1186/s13065-025-01584-1)

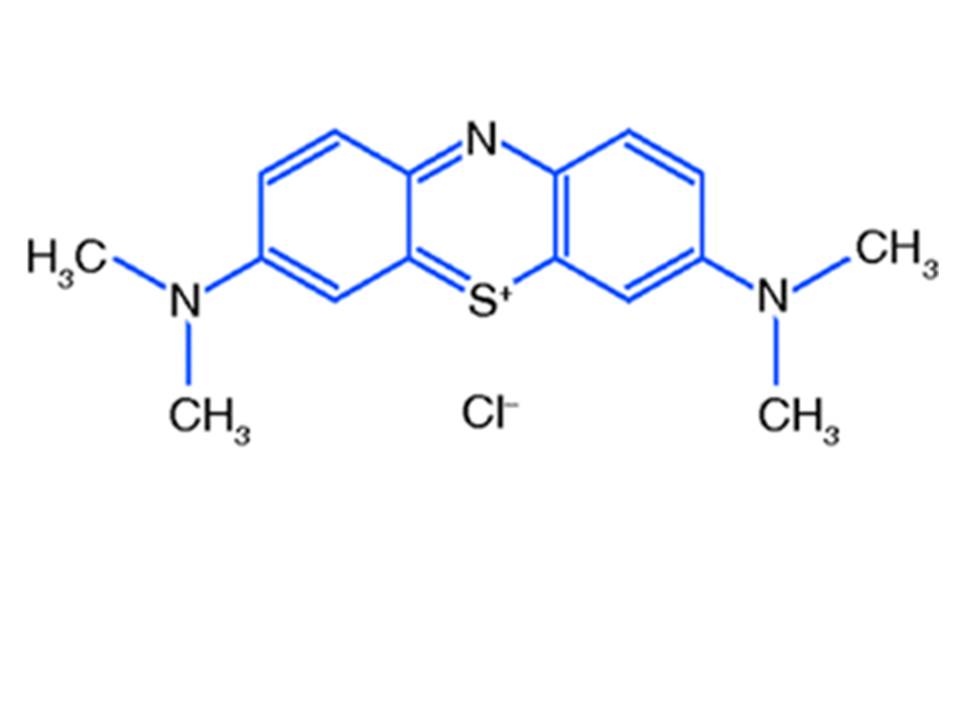

Supplement: Supplementary file 2 — Supplementary Material 2 [file 13065_2025_1584_MOESM2_ESM.jpg]

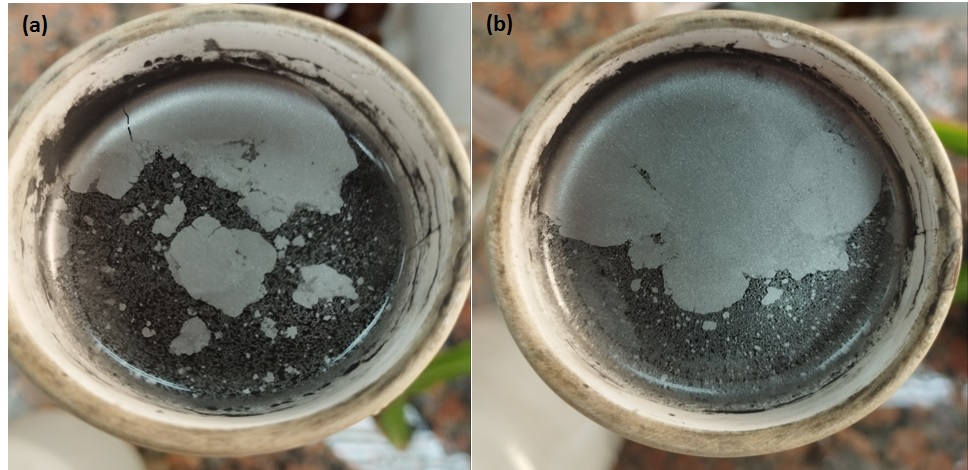

Supplement: Supplementary file 3 — Supplementary Material 3 [file 13065_2025_1584_MOESM3_ESM.jpg]
