# Supplementary material for: In situ decoration of Ag@exfoliated graphite composite catalyst for Fenton-like oxidation of methylene blue dye: kinetic and thermodynamic studies
Source: BMC Chem. 2025 Jul 24;19(1):221. doi: 10.1186/s13065-025-01584-1 (PMC12291263; doi:10.1186/s13065-025-01584-1)
Supplement: Supplementary file 5 — Supplementary Material 5 [file 13065_2025_1584_MOESM5_ESM.docx]

| **Table S1. BET analysis of prepared catalysts.** | | | | |
| --- | --- | --- | --- | --- |
| **Ag@EG (1:1)** | **Ag@EG (0.5:1)** | **EG** | **Textural Characteristics** |  |
| 87 | 75.4 | 53.1 | **S_BET_ (m^2^/g)** |  |
| 4.6 | 4.7 | 5.5 | **Mean pore**  **diameter (nm)** |  |
| 0.1 | 0.09 | 0.07 | **Total pore**  **volume (cm^3^/g)** |  |
